# Associations Between Complement Components and Vitamin D and the Physical Activities of Daily Living Among a Longevous Population in Hainan, China

**DOI:** 10.3389/fimmu.2020.01543

**Published:** 2020-07-17

**Authors:** Chi Zhang, Shihui Fu, Minghao Zhao, Deping Liu, Yali Zhao, Yao Yao

**Affiliations:** ^1^Department of Education, Beijing Hospital, National Center of Gerontology, Beijing, China; ^2^Institute of Geriatrics Medicine, Chinese Academy of Medical Sciences, Beijing, China; ^3^Department of Cardiology, Hainan Hospital of Chinese PLA General Hospital, Sanya, China; ^4^School of Medicine, Peking University Health Science Center, Beijing, China; ^5^Central Laboratory, Hainan Hospital of Chinese PLA General Hospital, Sanya, China; ^6^Center for Healthy Aging and Development Studies, National School of Development, Peking University, Beijing, China; ^7^Center for the Study of Aging and Human Development and Geriatrics Division, Medical School of Duke University, Durham, NC, United States

**Keywords:** complement C3, complement C4, vitamin D, physical activities of daily living, centenarians

## Abstract

**Background:** Vitamin D and complement components shared some common pathophysiological pathways in the musculoskeletal system, circulation, and metabolism, which were linked to physical function. It is hypothesized that serum complement components may interact with vitamin D in respect of the physical activities of daily living (PADLs).

**Objective:** To investigate if serum complement components 3 (C3), complement components 4 (C4), and 25-hydroxyvitamin D [25(OH)D] associate with PADLs, and to examine whether the association between 25(OH)D levels and PADLs varies at different complement component levels among Chinese centenarians.

**Methods:** This study was conducted in a group of population-based centenarians. PADLs were evaluated using the Barthel Index. Multiple regressions were used to analyze the associations among 25(OH)D, complements C3 and C4, and PADLs.

**Results:** Among 943 participants, 672 (71.3%) had physical dependence (PD). After adjusting for potential confounders, serum 25(OH)D and C3 levels were positively correlated with PADLs, while C4 levels were negatively correlated with PADLs (*P*s < 0.05). Serum 25(OH)D levels significantly interacted with both C3 (*P* for interaction = 0.033) and C4 (*P* for interaction = 0.006) levels on PADLs. At lower complement component levels, the multivariate odds ratios (ORs) of the upper tertile of vitamin D for PD were 0.32 (95% CI: 0.18–0.55) in the C3 group and 0.29 (95% CI: 0.16–0.50) in the C4 group. At higher complement component levels, the ORs in the C3 and C4 groups were not statistically significant.

**Conclusions:** In a group of population-based Chinese centenarians, we observed that serum complement C3 and 25(OH)D levels were positively associated with PADLs, while C4 was negatively associated with PADLs. The associations between 25(OH)D levels and PADLs were more pronounced in groups with lower serum complement component levels.

## Introduction

The complement system is an important constituent of the immune system, which is crucial for maintaining the health of older people (1). Complement components 3 (C3) and 4 (C4) are widely involved in three activating pathways of complements (classical pathway, alternative pathway, and mannose-binding lectin pathway) and they play vital roles in anti-infection and inflammatory response, as well as in metabolism and circulation regulation (2–4). Existing studies have shown that elevated levels of C3 and C4 were associated with higher prevalence or incidence of metabolic syndrome and cardiovascular diseases (4–9). In addition, the biological activity of the complement system is widely involved in muscle regulation (10–13). A recent experimental study has revealed the important role of C3 in the skeletal muscle regeneration in mice, by means of alternative pathway and C3a receptor (C3aR) signaling (14). Based on the physiological mechanisms of complement C3 and C4 mentioned above, they may be further associated with physical function. However, to the best of our knowledge, no human study with epidemiological or clinical designs have reported an association between the complement C3, C4, and the physical activities of daily living (PADLs).

Previous studies suggested that circulating 25-hydroxyvitamin D [25(OH)D], a major circulatory form of vitamin D (15), as an independent determinant for physical dependence (PD) (16–20) in older people, including centenarians (21). Pathophysiological mechanisms include its beneficial roles in skeletal muscle regeneration (22), arterial stiffness and endothelial function (23, 24), and immune regulation (25) etc. However, some other studies didn't demonstrate any association between impaired physical performance and low serum 25(OH)D levels (26–28). One plausible reason for these complex inconsistency results is the interaction of serum 25(OH)D with other biomarkers regarding to malnutrition or inflammation. For example, an epidemiological studies of the older over 60 years old in United States indicated interleukin-6 to be an important intermediary between vitamin D deficiency and chronic kidney disease (29). Another study in America showed that high C-reactive protein and low 25(OH)D levels were jointly associated with slow gait speed among individuals aged 50 and older (30). Our recent study also observed that the inverse association of serum 25(OH)D levels with all-cause mortality was only significant in subjects with higher albumin level (≥40 g/L), while the association failed to reach statistical significance in groups with lower albumin level (<40 g/L) (31). Additionally, the presence of inflammation also predicted a lower level of vitamin D; the serum 25(OH)D level was significantly associated with lower level of CRP and higher level of albumin (32). As routine immune biomarkers, elevated complement component 3 and 4 are commonly seen in infection, inflammation, or other immune-related pathological conditions (2, 3), and involved in muscle regulation and metabolism. Thus, serum complement components and vitamin D levels may exert interactive effects on physical function based on some common pathways mentioned above.

Maintenance of physical function in older people is important not only for healthy and independent lives in the community but also for preventing negative outcomes, such as disability and for early mortality (33–35). The role of serum complements C3, C4, and vitamin D as well as their interplays in the physical activity of people at advanced age is an intriguing research question, and of great importance for disability prevention and intervention. The current study was designed to investigate the association between serum complements C3, C4, 25(OH)D, and PADLs and to further test the hypothesis that whether the associations between serum 25(OH)D level and PADLs varies from different level of complement components.

## Methods

### Study Participants

**Figure 1:**
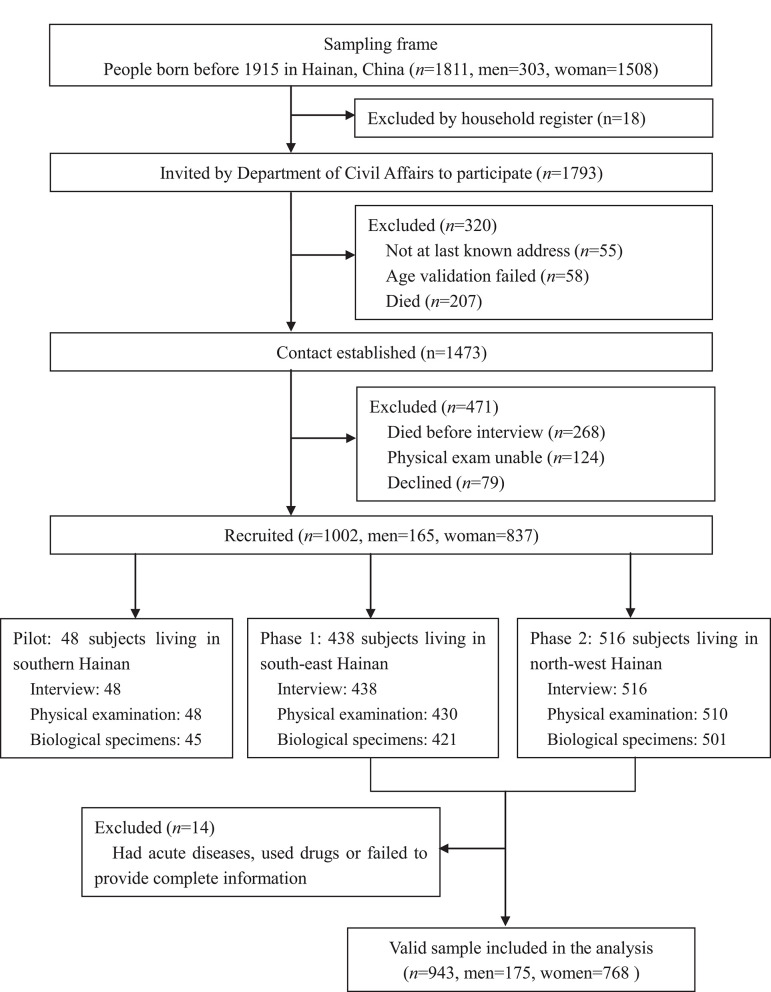
Flowchart of participants recruitment for China Hainan Centenarian Cohort Study (CHCCS). A total of 943 participants (173 males, 768 females) were included in the analysis.

The sample for this study was obtained from the China Hainan Centenarian Cohort Study (CHCCS), which was conducted in Hainan, China between June 2014 and December 2016. Details of this study, including sampling strategy and interview procedures, have been reported elsewhere (36, 37). A total of 1,002 centenarians (48 were involved in the pilot survey and 954 were involved in the formal survey) were interviewed at home or in community health service centers, following which, physical examinations and blood analyses were performed following standard procedures. After excluding 11 participants who had acute diseases, used drugs, or failed to provide complete information, 943 local individuals (175 men and 768 women, aged 100–115 years) from all 18 regions of Hainan Province were included in the final analysis (Figure 1). Prior to the investigation, three-step strict age verification methods were used to ensure the authenticity of the ages of the enrolled participants. First round: check with household registration, identification card, census-derived list. Second round: claimed birthday and Chinese zodiac sign, claimed age when they experienced specific social events. Third round: milestone assessments, such as marriage date, date of first-born child, subsequent birth dates of children, date of mother's death.

### Ethics

The CHCCS study was approved by the Ethics Committee of the Hainan Hospital of the Chinese People's Liberation Army General Hospital (Serial no. 301hn11201601). All participants or their legal representatives signed written consent forms in the survey. This study followed the Strengthening the Reporting of Observational Studies in Epidemiology (STROBE) reporting guidelines.

### Physical Activities of Daily Living (PADLs)

The Barthel Index of activities of daily living has been widely used to evaluate the physical function of centenarians (21, 38); the validity and reliability of this tool for use in the Chinese population of older people have been well-established (39). The Barthel Index consists of 10 items measuring a person's PADLs, such as grooming, feeding, dressing, bathing, toilet use, transferring from bed to chair, walking, stair climbing, bowel continence, and urinary continence. Each item of PADLs is rated on a structured scale, with a given number of points from 5 to 15 assigned to each level of activity, and the total score ranges from 0 to 100 points in 5-point increments (Table S1). A higher score indicates higher levels of physical function. The centenarians were defined as having PD when their total score was 90 points or less, otherwise the participants were regarded as having physical independence (PI) (40).

### Vitamin D and Complement C3 and C4 Status

Serum 25-hydroxyvitamin D [25(OH)D] is commonly measured to reflect vitamin D status (15). In this study, blood samples were obtained from each participant by experienced nurses and transported under cold storage to the Clinical Laboratory of the Hainan Hospital of the Chinese PLA General Hospital within 4 h. Serum 25(OH)D levels were measured by automated radioimmunoassay analyzers (DiaSorin, Stillwater, MN, USA) following a standard procedure. The inter-assay and intra-assay coefficients of variation for serum 25(OH)D levels in this study were 8.3 and 6.7%, respectively. Serum levels of complement components 3 and 4 were measured by the immunological scatting turbidity method using a fully automated protein analyzer (BNII; Siemens AG, Munich, Germany); the inter-assay and intra-assay coefficients of variation were 1.4–4.7% and 2.5–5.5%, respectively.

### Covariates

Home interviews employing standardized structured questionnaires were conducted in order to collect data on the demographic characteristics (age, gender, height, weight, ethnicity, education, marital, and birth status), season of blood collection, lifestyles (smoking, alcohol use, tea consumption, outdoor activities), and common physical conditions (hypertension, diabetes, dyslipidemia, visual, and auditory impairments) of the participants. Ethnicity was categorized as either “Han” (the predominant ethnicity in China) or “non-Han”; education level was categorized as “illiteracy” or not due to the generally poor level of education among centenarians in Hainan; the season of blood collection was dichotomized into summer (April–September) and winter (October–March). Hypertension, diabetes, and dyslipidemia were diagnosed according to the corresponding biochemical indicators (6); depressive symptoms were measured by the shortened version of the Geriatric Depression Scale (GDS-15) (41). Current health status and medical history, including visual impairment and auditory impairment, were recorded as self-reported combined with the medical records of the participants.

Clinical examinations were conducted by experienced nurses. Height (H), weight (W), waist circumference (WC), and hip circumference (HC) were measured using a standard scale with participants barefoot and dressed in light clothing (Seca, Germany). Each parameter was measured twice, and the reported results were the averages of these duplicate measurements. Body mass index (BMI) was calculated as the weight in kilograms divided by the square of the height in meters. Systolic and diastolic blood pressures (SBP and DBP) were measured twice using electronic sphygmomanometers (Omron Hem-7200, Japan) consecutively, with at least a 1 min interval between measurements, and the reported blood pressures were the averages of the two measurements.

Samples of venous blood were extracted from the centenarians and transported within 4 h in bio-transport containers (4°C) to a central laboratory in Hainan Hospital of the Chinese PLA General Hospital. Serum levels of total cholesterol (TC), fasting blood glucose (FBG), hemoglobin (HB), and creatinine (CRE) were measured using enzymatic assays (Roche Products Ltd., Basel, Switzerland) on a fully automated biochemical autoanalyzer (COBAS c702; Roche Products Ltd., Basel, Switzerland). Estimated glomerular filtration rate (eGFR) was calculated using a modified version of the Modification of Diet in Renal Disease (MDRD) equation based on data from Chinese patients as follows: 175 × serum creatinine (mg/dL) - 1.234 × age (years) - 0.179 × 0.79 (if female) (42). Other immunological factors, such as immunoglobulin A, E, G, and M and C-reactive protein (CRP), were measured with a fully automated protein analyzer (BNII; Siemens AG, Munich, Germany).

### Statistical Analysis

Continuous variables were described as the mean ± SD; categorical variables were described as numbers and percentages. Continuous variables were compared using the Student's *t*-test (normal distribution) or the Wilcoxon rank-sum test (skewed distribution); categorical variables were compared using the chi-squared test. Multiple linear regressions were used to analyze the correlations between vitamin D, C3, C4, and PADLs. In the subsequent analysis, serum 25(OH)D levels were categorized into tertiles (low, ≤18.3 ng/mL; 18.3 ng/mL< intermediate ≤25.6 ng/mL; high, >25.6 ng/mL), and the low stratum was defined as the reference group. Levels of C3 and C4 had a skewed distribution and were divided into two categories by medians (97.0 mg/dL for C3, and 22.8 mg/dL for C4). Multiple logistic regressions were implemented to analyze the associations between C3, C4, and vitamin D as well as their interaction with PD. The significance of multiplicative interaction between 25(OH)D and C3, C4 for PD was tested by adding cross-product terms in the models. Model 1 was unadjusted; Model 2 was adjusted for sex, age, BMI, education, and smoking and drinking habits; Model 3 was further adjusted for depressive syndromes, visual and auditory impairments, SBP, DBP, FBG, TC, eGFR, CRP, and season of blood collection. The missing data were filled in using the mean value or multiple imputation method. Statistical significance was accepted at the two-sided 0.05 level, and confidence intervals were computed at the 95% level. Statistical analyses were performed with SPSS Statistics (version 22.0 for Windows; IBM Corporation, Armonk, NY, USA).

## Results

### Baseline Characteristics

The study sample included 768 women (81.4%) and 175 men (18.6%), with a mean age of 102.9 ± 2.76 years. The prevalence of PD in the total sample was 71.3% (74.1% of women and 58.9% of men, *P* < 0.001). The means ± standard deviations of baseline serum 25(OH)D were 21.8 ± 9.5 g/L for the PD group and 25.2 ± 8.9 g/L for the PI group (*P* < 0.001). There were no individuals with clinically abnormal high serum 25(OH)D levels (>100 g/L) in either gender groups. The means ± standard deviations of baseline serum complement C3 were 99.6 ± 21.3 mg/dL for the PD participants and 100.0 ± 23.5 mg/dL for the PI participants (*P* = 0.788). The means ± standard deviations of baseline serum complement C4 were 24.4 ± 8.5 mg/dL for the PD participants and 23.8 ± 8.6 mg/dL for the PI participants (*P* = 0.298). Individuals with either clinically abnormal serum complement C3 (>180 mg/dL) and C4 (>50 mg/dL) were rare among the centenarians (1 man for C3; 8 women for C4). Table 1 summarizes the general characteristics of participants with and without PD.

**Table 1:** Characteristics of participants according to physical activities of daily living (PADLs).

**Characteristics**	**Overall (*n* = 943)**	**Physical independency (*n* = 271)**	**Physical dependency (*n* = 672)**	***P*-value**
Female, %	81.4%	73.4%	84.7%	<0.001
Age, y	102.9 ± 2.76	102.6 ± 2.7	102.9 ± 2.8	0.174
Barthel Index score	74.85 ± 24.87	97.65 ± 2.50	65.67 ± 23.91	<0.001
Complement C3, mg/dL	99.7 ± 21.9	100.0 ± 23.5	99.6 ± 21.3	0.788
Complement C4, mg/dL	24.2 ± 8.6	23.8 ± 8.6	24.4 ± 8.5	0.298
25(OH)D, ng/mL	22.7 ± 9.4	25.2 ± 8.9	21.8 ± 9.5	<0.001
BMI, kg/m^2^	18.2 ± 3.2	19.1 ± 3.2	17.9 ± 3.2	<0.001
Illiteracy, %	91.0%	88.2%	92.1%	0.060
Current smoking, %	3.51%	3.40%	3.64%	0.974
Alcohol consumption, %	10.64%	8.11%	13.90%	0.004
Visual impairment, %	28.0%	15.5%	33.0%	<0.001
Auditory impairment, %	31.2%	21.8%	35.0%	<0.001
Depressive syndrome, %	30.9%	17.7%	36.2%	<0.001
SBP, mmHg	153.2 ± 25.1	153.7 ± 24.3	151.8 ± 25.4	0.297
DBP, mmHg,	75.4 ± 13.3	75.9 ± 12.9	75.1 ± 13.4	0.514
FBG, mmol/L	5.20 ± 1.50	5.00 ± 1.48	5.22 ± 1.51	0.049
TC, mmol/	4.71 ± 1.03	4.76 ± 0.97	4.64 ± 1.02	0.107
eGFR, ml/min/1.73 m^2^	68.8 ± 24.0	66.0 ± 22.0	69.9 ± 24.6	0.025
CRP, mg/dL	0.63 ± 2.37	0.55 ± 3.72	0.66 ± 1.53	0.483
Season of blood collection, summer^a^	65.4%	68.6%	64.1%	0.199

a *Summer: April–September; P-values are based on the Student's t-test, Wilcoxon rank-sum test, or chi-squared test*.

*25(OH)D, 25-hydroxyvitamin D; BMI, body mass index; complement C3, complement component 3; complement C4, complement component 4; CRP, C-reactive protein; DBP, diastolic blood pressure; eGFR, estimated glomerular filtration rate; FBG, fasting blood glucose; SBP, systolic blood pressure; TC, total cholesterol*.

### Associations of Serum Complements C3 and C4 and Vitamin D Levels With PADLs

**Figure 2:**
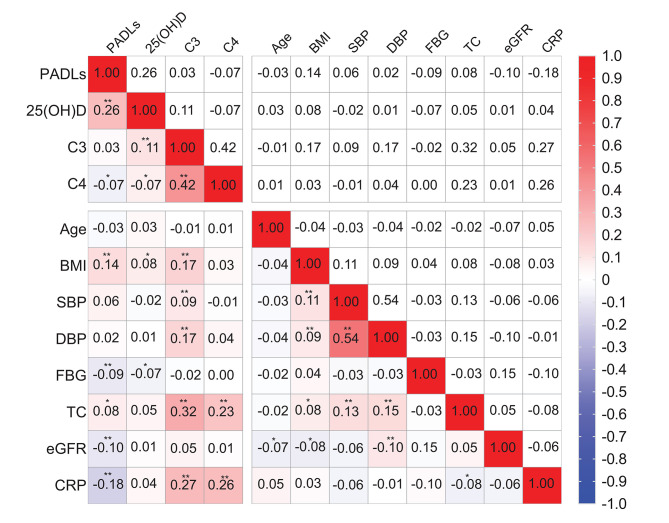
Spearman correlation coefficients between physical activities of daily living (PADLs), 25(OH)D, complement C3, C4, and other covariates. *P < 0.05 and **P < 0.01. 25(OH)D, 25-hydroxyvitamin D; BMI, body mass index; complement C3, complement component 3; complement C4, complement component 4; CRP, C-reactive protein; SBP, systolic blood pressure; DBP, diastolic blood pressure; eGFR, estimated glomerular filtration rate; FBG, fasting blood glucose; TC, total cholesterol.

Figure 2 showed the correlation coefficients of C3, C4, vitamin D, and Barthel score as well as other covariates. Serum 25(OH)D was positively correlated with Barthel score (*r* = 0.26, *P* < 0.01), C4 was negatively correlated with Barthel score (*r* = −0.068, *P* < 0.05), and no significant correlation was found between C3 and PADLs (*r* = 0.029, *P* = 0.375). The associations of serum complements C3 and C4 and vitamin D levels with PADLs are presented in Table 2. After adjusting for all confounders (Model 3), serum 25(OH)D (β = 0.53, *P* = 0.006) and C3 levels (β = 0.11, *P* = 0.008) were positively associated with the PADLs scores, while C4 levels (β = −0.29, *P* = 0.003) were negatively associated with the PADLs scores. In addition, both C3 (*P* for interaction = 0.033) and C4 (*P* for interaction = 0.006) were significantly associated with serum vitamin D for PD, and the interaction was more pronounced and solid between C4 and vitamin D (Tables S2, S3).

**Table 2:** Association between serum complements C3 and C4 and vitamin D levels and PADLs.

**Feature**	**Model 1**				**Model 2**				**Model 3**			
	**β**	***SE***	***T***	***P*-value**	**β**	***SE***	***T***	***P*-value**	**β**	***SE***	***T***	***P*-value**
C3, mg/dL	0.05	0.04	1.23	0.217	0.07	0.04	1.83	0.068	0.11	0.04	2.64	0.008
C4, mg/dL	−0.18	0.09	1.86	0.064	−0.30	0.10	3.04	0.002	−0.29	0.10	3.01	0.003
25(OH)D, g/mL	0.61	0.08	7.26	<0.001	0.56	0.09	6.40	<0.001	0.53	0.09	2.77	0.006

*25(OH)D, 25-hydroxyvitamin D; C3, complement component 3; C4, complement component 4; PADLs, physical activities of daily living*.

*Model 1: unadjusted; Model 2: adjusted for sex, age, BMI, education, smoking, and drinking habits; Model 3: further adjusted for depressive syndromes, visual and auditory impairments, SBP, DBP, FBG, TC, eGFR, CRP, and season of blood collection*.

### Interaction Between Serum Complements C3 and C4 and Vitamin D Levels for PD

**Figure 3:**
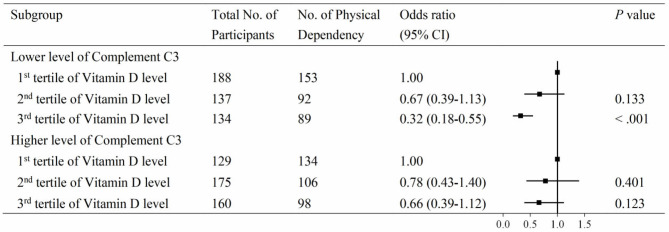
Associations between vitamin D and physical disability according to compliment C3 levels. Serum 25(OH)D levels were categorized into tertiles (low, ≤18.3 ng/mL; intermediate, 18.3–25.6 ng/mL; high, >25.6 ng/mL), and the low stratum was defined as the reference group. Levels of compliment C3 had a skewed distribution and were divided into two categories by medians (cutoff = 97.0 mg/dL).

**Figure 4:**
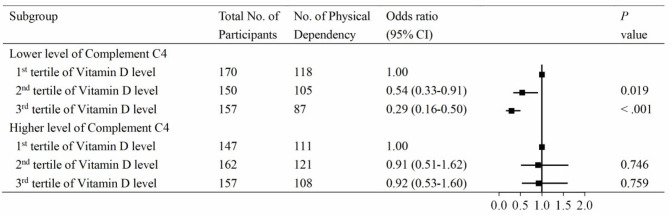
Associations between vitamin D and physical disability according to compliment C4 levels. Serum 25(OH)D levels were categorized into tertiles (low, ≤18.3 ng/mL; intermediate, 18.3–25.6 ng/mL; high, >25.6 ng/mL), and the low stratum was defined as the reference group. Levels of compliment C4 had a skewed distribution and were divided into two categories by medians (cutoff = 22.8 mg/dL).

To further investigate the association between vitamin D and PD according to the levels of C3 and C4, serum 25(OH)D levels were divided into three groups by tertiles, with the lowest group as the reference. The associations between vitamin D and PD according to the level of C3 are shown in Figure 3. At the lower level of complement C3, the multivariable adjusted odds ratio (OR) of the third tertile of vitamin D for PD was 0.32 (95% CI: 0.18–0.55). Similar results were observed in the C4 group (Figure 4). At the lower level of complement C4, the multivariable OR of the second tertile of vitamin D for PD was 0.54 (95% CI: 0.33–0.91), and that of the third tertile was 0.29 (95% CI: 0.16–0.50). However, neither of the above two ORs was statistically significant in the higher C4 group. The tendencies in the C3 and C4 groups were basically consistent, and the results were not substantially altered when removing those participants with clinically abnormal C3 and C4 levels.

## Discussion

For the first time, as far as we are aware, this study has explored the association of serum 25(OH)D, complement C3 and C4 levels with PADLs, as well as their interactions among a longevous population in Hainan Province, China. Our major findings were that serum 25(OH)D and complement C3 levels were positively associated, while complement C4 was negatively associated with PADLs among this population. However, the association between C3 and PADLs only reach statistical significance in full-adjusted model. We also observed that the associations between serum 25(OH)D and PD were significantly varied at different complement component C3 and C4 levels; the associations were more pronounced in groups with lower levels of serum C3 or C4, but lost statistical significance in higher complement component groups.

Few studies have focused on the relationships between the complement system and PADLs, although these relationships shared common biological pathways. Our result from full-adjusted model showed that complement C3 levels were positively associated with PADLs, while complement C4 levels were negatively associated with PADLs. Different involvements in the three activation complement pathways (classical pathway, alternative pathway, and mannose-binding lectin pathway) may partly explain the opposite associations of C3 and C4 with PADLs in our study. According to Zhang's study, complement C3 activates cardiotoxin-injured muscle regeneration, which is critical for maintaining muscle mass and movement during the aging process and following injury (14). The mechanism is such that C3a–C3aR signaling recruits monocytes/macrophages to infiltrate and then produces growth factors and cytokines to promote the proliferation and differentiation of myoblasts (43). This process works through alternative pathways rather than through classical or lectin pathways, while C4 is involved mainly in classical and lectin pathways but not in alternative pathways. The complement component, as an important inflammatory mediator and immune marker in human bodies, is linked to metabolic and cardiovascular disorders or some other inflammation-related pathological states (44, 45), which explains the negative correlation between C4 and PADLs. However, as for C3, its effect on PADLs can be considered as two-fold. As an immune-inflammatory mediator, it plays a similar role to C4 and we have confirmed the significant association between complement system and metabolic syndrome in Chinese community-dwelling centenarians in our previous study (6). In contrast, C3 also plays a key role in promoting skeletal muscle regeneration in the process of aging and injury according to previous experimental studies. Therefore, although we have obtained epidemiological evidence suggesting that the levels of C3 in centenarians significantly associated with physical function, but the positively association between C3 and PADLs was not steady and the significance verified with different adjusted models. In model 3, we adjusted for variables related to renal function, endocrine, and immune states (FBG, eGFR, and CRP, etc.), which might account for the statistical significance between complement component 3 and PADLs. Thus, the specific mechanism of complement system on physical function needs to be verified by investigations among different older populations as well as in experimental studies.

Several studies have explored the physiological interplay between serum 25(OH)D and immunological biomarkers in humans. A population-based cross-sectional study of 1,826 participants aged 50–85 years in the USA found that high C-reactive protein and low 25(OH)D levels were associated with slow gait speed (30). Our recent study observed a similar trend that the inverse association of serum 25(OH)D levels with all-cause mortality was only significant in subjects with higher albumin level (≥40 g/L), while the association failed to reach statistical significance in groups with lower albumin level (<40 g/L) (31). Although many fundamental and epidemiological studies have explored how vitamin D affects physical function, little is known about its synergistic effects involving inflammatory biomarkers. The interaction between serum 25(OH)D and C3/C4 found in this study can be explained in terms of the three action pathways of vitamin D on PADLs.

In the first action pathway, vitamin D acts directly on skeletal muscle cells by accelerating the entry of Ca^2+^ ions into bone tissue through the osteoblast cell membrane, which promotes bone salt deposition, increases bone calcium storage, and enhances muscle contraction (46). Furthermore, vitamin D can also act on the nucleus of intestinal mucosal cells, promotes the biosynthesis of calcium transporter, accelerates combination with calcium into a soluble complex, induces absorption of calcium, and promotes absorption of phosphorus through the calcium and phosphorus balance mechanism (47). Therefore, the above two aspects suggest that vitamin D plays an important role in maintaining the balances between blood calcium and bone calcium, calcium and phosphorus, and promoting bone calcification and bone growth. Many recent studies have reported that elevated complement C3 and C4 levels are risk factors for metabolic syndrome (5, 7, 8) and cardiovascular diseases (9), while vitamin D is a protective factor (48–50). Vitamin D can prevent vascular calcification and atherosclerosis (23) by regulating the expression of vascular smooth muscle cells (51) and the function of endothelial cells (24). In addition, the human circulatory system and blood flow dynamics are thwarted under conditions of atherosclerosis and coronary heart disease, the results of which are that the decreased serum calcium transported to skeletal muscle cells affects the blood and bone calcium balance and inhibits the promotion of vitamin D transported to the skeleton.

In the second pathway, the complement system has important properties of immune protection and resistance to inflammation. Increased C3 and C4 levels are commonly seen in autoimmune dysfunction, tissue damage, and related inflammatory diseases. Their levels are closely related to coronary syndrome (52), rheumatoid arthritis (53), and other immunological diseases. The concentrations of vitamin D and immune factors are also associated. Previous studies have shown that vitamin D increases the production of some anti-inflammatory cytokines and reduces the release of some pro-inflammatory cytokines (54, 55). Some studies have found that patients with inflammatory bowel-related disease have an impaired vitamin D status (56, 57). Systemic inflammation reduced the concentrations of 25(OH)D in patients who underwent primary knee arthroplasty (58). Accordingly, a lack of vitamin D induces a nuclear factor-related inflammatory reaction (59, 60) and is associated with inflammation-related disability (61). Moreover, vitamin D replacement can normalize the levels of inflammatory marker in individuals with congestive heart failure (62). As for the role of vitamin D, it can enhance individuals' ability to fight infection by regulating the innate and adaptive immune function, which is also one of the physiological pathways to promote physical function (25, 63). Thus, vitamin D and C3 and C4 levels have a potential synergetic effect in immune and inflammatory pathways. Although the specific mechanism is complex and involves the pathogenesis of different diseases, it can partly be explained by stating that the immune or inflammation-related pathological state, reflected by high levels of complement components, may antagonize the role of vitamin D.

In the third pathway, in terms of the metabolism of vitamin D, the liver and kidneys are the two most important organs. Vitamin D3 is hydroxylated to 25(OH)D in the endoplasmic reticulum and mitochondria of hepatocytes and then activated again by the 1-hydroxylase system to 1,25-(OH)2D3 in the epithelial cells of the proximal convoluted tubule of the kidney (64). C3 and C4 are closely related to liver (65) and kidney diseases (66), such as hepatitis B and C, IgA nephropathy, and glomerulonephritis, respectively. Therefore, levels of complement components can reflect liver and kidney function to a certain extent. Meanwhile, activation and reabsorption of vitamin D may also be inhibited under the pathological conditions related to high C3 or C4 levels. Given the long half-life and stability of 25(OH)D (67), we analyzed 25(OH)D as a proxy for vitamin D levels, as most studies have done (15). At higher levels of C3 or C4, the association between vitamin D and PD was not significant, which might be caused by the low levels of 1,25-(OH)2D3 following activation. This explanation needs to be verified by more detailed measurements in future studies.

Several observational studies have reported associations between functional decreases and lower serum 25(OH)D levels (20, 68), including a few randomized control trials (18, 69), while some vitamin D interventional studies have not yielded satisfactory results (70). The factors that influence the relationship between vitamin D levels or supplements and functional improvement are complex, and interactions between vitamin D and other biochemical indicators may partly account for those negative results. Our findings on the interaction between vitamin D and complements components not only help explain the inconsistent association of vitamin D and PADLs, but also have potentially significant implications for interventional strategies to improve the health of older adults. The priority of intervention on physical decline could be immunological measures to increase levels of complement components that would occur in combination with vitamin D supplementation. In addition, it might be useful for physical function to maintain adequate vitamin D levels when comes with the low levels of complement components. In summary, for those with vitamin deficiency, complement components screening and immunological counseling might be of help before implementing any vitamin D interventions.

Several limitations of the current study should be acknowledged. Firstly, the results did not indicate any causal inferences due to the cross-sectional design, and the extrapolation to other populations requires further validation. Secondly, the current health status of individuals potentially confounded the results due to its being collected by self-report, and other confounders such as cognitive impairment were not included in this study. Thirdly, as the current household registration system did not exist in China a 100 years ago, the ages of longevous persons based on the Chinese identity card number (Resident Identity Card), which was officially introduced in the 1980s, could not be properly validated due to a lack of solid evidence. Nevertheless, very strict quality control measures have been taken to ensure the authenticity of participants' ages.

In conclusion, serum complement C3 and 25(OH)D levels were positively associated with PADLs, while C4 was negatively associated with PADLs among Chinese centenarians. The associations between 25(OH)D levels and PADLs were more pronounced in groups with lower levels of complement component C3 and C4. Our findings imply that the physical intervention potential of intrinsic vitamin D, may, in part, depend upon individual inflammatory profiles. If substantiated by future interventional studies, our results suggest the value of immunological screening for complement components for more precise and potentially more effective vitamin D interventional measures on PADLs.

## Data Availability Statement

The data analyzed in this study was subject to the following licenses/restrictions: the datasets analyzed in the current study are not publicly available due to the personal privacy of the participants but are available from Yali Zhao upon request. Requests to access these datasets should be directed to Yali Zhao, zhaoyl301@163.com.

## Ethics Statement

Each participant provided written informed consent before the study and the Ethics Committee of the Hainan Hospital of the Chinese People's Liberation Army General Hospital (Sanya, Hainan) approved the study protocol (No. of serial: 301hn11201601).

## Author Contributions

CZ, YZ, and YY proposed the concept and design, analyzed and interpreted the data, and wrote the manuscript. CZ, SF, YZ, and YY interpreted the data, drafted and edited the manuscript, supervised the study, and obtained funding. MZ and DL drafted and edited the manuscript. YY and YZ were guarantors. All authors read and approved the final version of the manuscript.

## Conflict of Interest

The authors declare that the research was conducted in the absence of any commercial or financial relationships that could be construed as a potential conflict of interest.

## Abbreviations

25(OH)D: 25-hydroxyvitamin D3
BMI: body mass index
complement C3: complement component 3
complement C4: complement component 4
CRP: C-reactive protein
SBP: systolic blood pressure
DBP: diastolic blood pressure
eGFR: estimated glomerular filtration rate
FBG: fasting blood glucose
PADLs: physical activities of daily living
PD: physical dependence
PI: physical independence
SBP: systolic blood pressure
TC: total cholesterol.
