# Supplementary material for: Associations Between Complement Components and Vitamin D and the Physical Activities of Daily Living Among a Longevous Population in Hainan, China
Source: Front Immunol. 2020 Jul 17;11:1543. doi: 10.3389/fimmu.2020.01543 (PMC7379858; doi:10.3389/fimmu.2020.01543)
Supplement: Supplementary file 1 [file Table_1.DOCX]

**Associations between complement components and vitamin D and the physical activities of daily living among a longevous population in Hainan, China**

Chi Zhang, Shihui Fu3, Minghao Zhao, Deping Liu, Yali Zhao, and Yao Yao

**Table S1…………………………………………………………………………………… Page 2**

Rating scale of activities of daily living (ADLs) according to the Barthel Index.

**Table S2…………………………………………………………………………………… Page 3**

Component C3 and its interactions with vitamin D for physical dependency.

**Table S3 …………………………………………………………………………………... Page 4**

Component C4 and its interaction with vitamin D for physical dependency.

**Table S1.** Rating scale of activities of daily living (ADLs) according to the Barthel Index

| Task | Rating | Task | Rating |
| --- | --- | --- | --- |
| Bathing | 0 = dependent  5 = independent | Grooming | 0 = needing help for personal care  5 = independent |
| Feeding | 0 = unable  5 = needing help for cutting and spreading food or requiring modified diet  10 = independent | Dressing | 0 = dependent  5 = needing help, but can do about half unaided  10 = independent (including buttons, zips, and laces) |
| Bowel control | 0 = incontinent or needing to be given enemas  5 = occasional accident  10 = continent | Urinary control | 0 = incontinent or catheterized and unable to manage alone  5 = occasional accident  10 = continent |
| Toilet use | 0 = unable  5 = needing some help, but can do something alone  10 = independent (on and off, dressing, wiping) | Stair climbing | 0 =unable  5= needing help (verbal, physical, carrying aid)  10 = independent |
| Transferring from bed to chair | 0 = unable, no sitting balance  5 = major help (one or two people), can sit  10 = minor help (verbal or physical)  15 = independent | Walking (on level surface) | 0 = immobile or <20 m  5 = wheelchair independent, >20 m  10 = walking with the help of one person, >20 m  15 = independent (but may use aid such as a stick) >20 m |

**Table S2.** Component C3 and its interactions with vitamin D for physical dependency

| Feature | Model 1 | | | |  | Model 2 | | | |  | Model 3 | | | |
| --- | --- | --- | --- | --- | --- | --- | --- | --- | --- | --- | --- | --- | --- | --- |
|  | *β* | *SE* | *T* | *P* value |  | *β* | *SE* | *T* | *P* value |  | *β* | *SE* | *T* | *P* value |
| C3 > 97.0 mg/dL | −0.79 | 0.54 | 2.13 | 0.144 |  | −0.78 | 0.56 | 1.94 | 0.163 |  | −0.86 | 0.57 | 2.27 | 0.132 |
| 25(OH)D< 20 ng/mL | 1.61 | 0.49 | 11.00 | 0.001 |  | 1.60 | 0.50 | 10.03 | 0.002 |  | 1.67 | 0.52 | 10.42 | 0.001 |
| C3×25(OH)D | −0.61 | 0.31 | 3.77 | 0.052 |  | −0.65 | 0.32 | 4.08 | 0.043 |  | −0.70 | 0.33 | 4.54 | 0.033 |

Abbreviations: 25(OH)D: 25-hydroxyvitamin D; C3: complement component 3.

Model 1: unadjusted; Model 2: adjusted for sex, age, BMI, education, smoking and drinking habits; Model 3: further adjusted for depressive syndromes, visual and auditory impairments, SBP, DBP, FBG, TC, eGFR, CRP, and season of blood collection.

**Table S3.** Component C4 and its interaction with vitamin D for physical dependency

| Feature | Model 1 | | | |  | Model 2 | | | |  | Model 3 | | | |
| --- | --- | --- | --- | --- | --- | --- | --- | --- | --- | --- | --- | --- | --- | --- |
|  | *β* | *SE* | *T* | *P* value |  | *β* | *SE* | *T* | *P* value |  | *β* | *SE* | *T* | *P* value |
| C4 > 22.8 mg/dL | 1.19 | 0.54 | 4.75 | 0.029 |  | 1.22 | 0.56 | 4.72 | 0.030 |  | 1.28 | 0.58 | 4.99 | 0.025 |
| 25(OH)D <20 ng/mL | 1.93 | 0.50 | 15.1 | <0.001 |  | 1.89 | 0.52 | 13.48 | <0.001 |  | 1.97 | 0.53 | 13.90 | <0.001 |
| C4×25(OH)D | −0.82 | 0.31 | 6.91 | 0.009 |  | −0.85 | 0.32 | 7.01 | 0.008 |  | −0.91 | 0.33 | 7.56 | 0.006 |

Notes: 25(OH)D: 25-hydroxyvitamin D; C4: complement component 4.

Model 1: unadjusted; Model 2: adjusted for sex, age, BMI, education, smoking and drinking habits; Model 3: further adjusted for depressive syndromes, visual and auditory impairments, SBP, DBP, FBG, TC, eGFR, CRP, and season of blood collection.
